# Methamphetamine abuse and “meth mouth” in Europe

**DOI:** 10.4317/medoral.20204

**Published:** 2015-02-07

**Authors:** Carlo De-Carolis, Geraldine-A. Boyd, Luca Mancinelli, Stefano Pagano, Stefano Eramo

**Affiliations:** 1 DDS. Department of Surgical and Biomedical Sciences-School of Dentistry- University of Perugia, Italy; 2 Language Centre (CLA), University of Perugia, Italy; 3 Geology Department, University of Dublin, Ireland

## Abstract

With easy chemical synthesis from its precursor, methamphetamine (MA) is now widespread in many countries. The abuse of methamphetamine is associated with several negative effects on health, because MA is a neurotoxin and a dangerous central nervous system stimulant. It changes levels of neurotransmitters in the brain, releasing dopamine and inhibiting nor epinephrine uptake which increases sympathetic nervous system activity and can lead to cardiac arrhythmia, hypertension and tachypnea. The consequences of MA abuse are clearly manifested in oral diseases (like “meth mouth”) which is characterised by extensive caries, teeth grinding with ensuing dental wear and trismus. The present review was designed to fill the gap in knowledge about methamphetamine abuse in the European Union (EU) and to illustrate the main clinical effects of prolonged use. After describing the pharmacology and systemic effects of methamphetamine and concentrating on its effects on the mouth, the present review compares the epidemiology and incidence of abuse in the world, particularly the USA and the EU.

** Key words:**Methamphetamine, “Meth mouth”, drug abuse, oral health.

## Introduction

Methamphetamine (MA) belongs to the amphetamine-type stimulant (ATS) class of substances which includes two categories: drugs like amphetamine, methamphetamine and their correlates such as fenetylline, methylphenidate, phenmetrazine, cathinone and so on and “ecstasy-type” drugs like MDMA, MDA and MDEA (1).

MA is a colourless, insoluble volatile oil. As a hydrochloride salt it is a bitter tasting, crystalline white powder that is easily dissolved in water or alcohol and often used today as an illegal drug. Although it may be inhaled or injected, it is usually smoked, swallowed as a pill or dissolved in a drink because of its bitter taste. Popular names for methamphetamine in powder are: Crank, Crypto, Fire, Meth, Speed. MA powder needs to be purified into a large crystal to be smoked. Hence the names: Crystal Meth, Crystal Glass, Ice (2).

More powerful and easier to produce than amphetamine, MA was first synthesized in Japan in 1919 , and was widely used by all sides in World War II to keep the troops in a state of constant alertness. Indeed, Japanese kamikaze pilots received high doses before their suicide missions (3). After various ups and downs (see  Table 1), MA rapidly became popular in the USA in the 1990s and was introduced into Europe through the Czech Republic (1). It gives a false sense of well-being, energy and over-estimation of physical and mental capacities and leads to a state of collapse once its effect is over. Prolonged use leads to addiction in a series of stages which are characteristically described as the (2,4): “rush” i.e. the effect of smoked or injected MA which is associated with tachycardia, sweating and increased blood pressure for about 30 minutes; “high”, i.e. a hyper-reactive, arrogant attitude lasting for several hours; “binge” i.e. a long period of 3 to 15 days during which the addict tries to maintain a constant “high” by ingesting higher and higher MA doses; “tweaking” or “itch” which develops at the end of a binge, when high MA doses no longer produce a “rush” or a “high”. This addiction stage is associated with skin hyperesthesia and tactile hallucinations which mimic the presence of insects under the skin. The addict suffers from psychosis, insomnia, persecution mania and neglects personal hygiene.

**Table 1:**
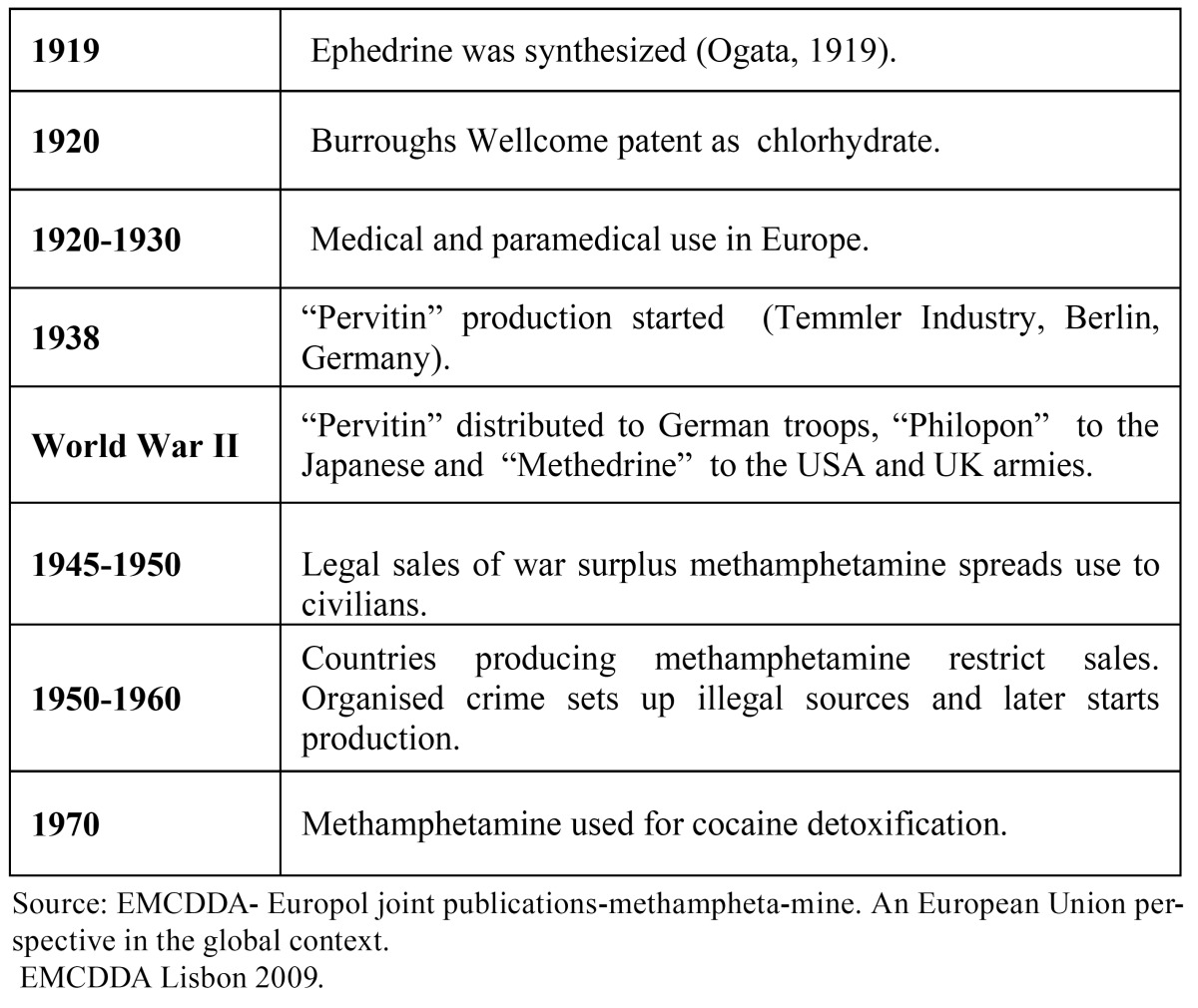
History of methamphetamine.

In the final stages of addiction, when new doses cannot be acquired, a few days lethargy is followed by recovery characterized by organic wasting which prevents normal activities. Finally, after one or two months the addict experiences the onset of withdrawal symptoms with depression, dysphoria, fatigue and suicidal ideation.

MA abuse can also be summarized in three escalating stages.

- Occasional or low-intensity use. The individual ingests MA pills or inhales powder to achieve “highs” or weight loss.

- Uncontrolled use. The individual smokes or inject MA to achieve rapid, intense effects, triggering essentially psychological addiction.

- High-intensity use. The individual (“speed freak”) is psychologically and physically addicted, demanding higher and higher doses.

Abuse of MA is associated with several negative effects on health, particularly “Meth mouth” which is characterized by widespread caries, teeth grinding with ensuing dental wear, and lockjaw (2,4). Since almost all reports to date have focused on the links between MA abuse and oral damages in the USA, the present review was designed to fill the gap in knowledge about MA abuse in European Union (EU) countries. After describing the pharmacology and systemic effects of MA, concentrating on its effects on the mouth, the present review compares epidemiology and incidence of abuse in the USA and EU.

## Pharmacology

**Figure 1:**
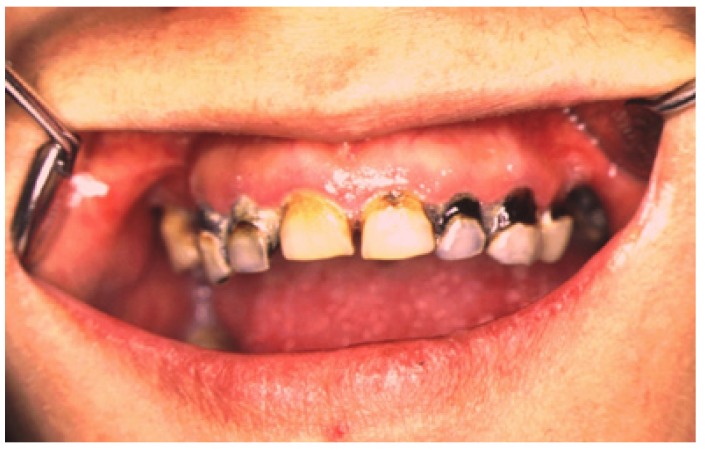
A clinical case of initial “meth mouth” in a young drug addict.

Figure 1 and  Table 2 show the formula and chemical properties of MA which is a neurotoxin and, even in small doses, a powerful central nervous system stimulant. It changes levels of monoamines and neurotransmitters in the brain, releasing dopamine and inhibiting nor-epinephrine uptake which increases activity of the sympathetic nervous system and can lead to cardiac arrhythmia, hypertension and tachypnea (5,6).

**Table 2:**
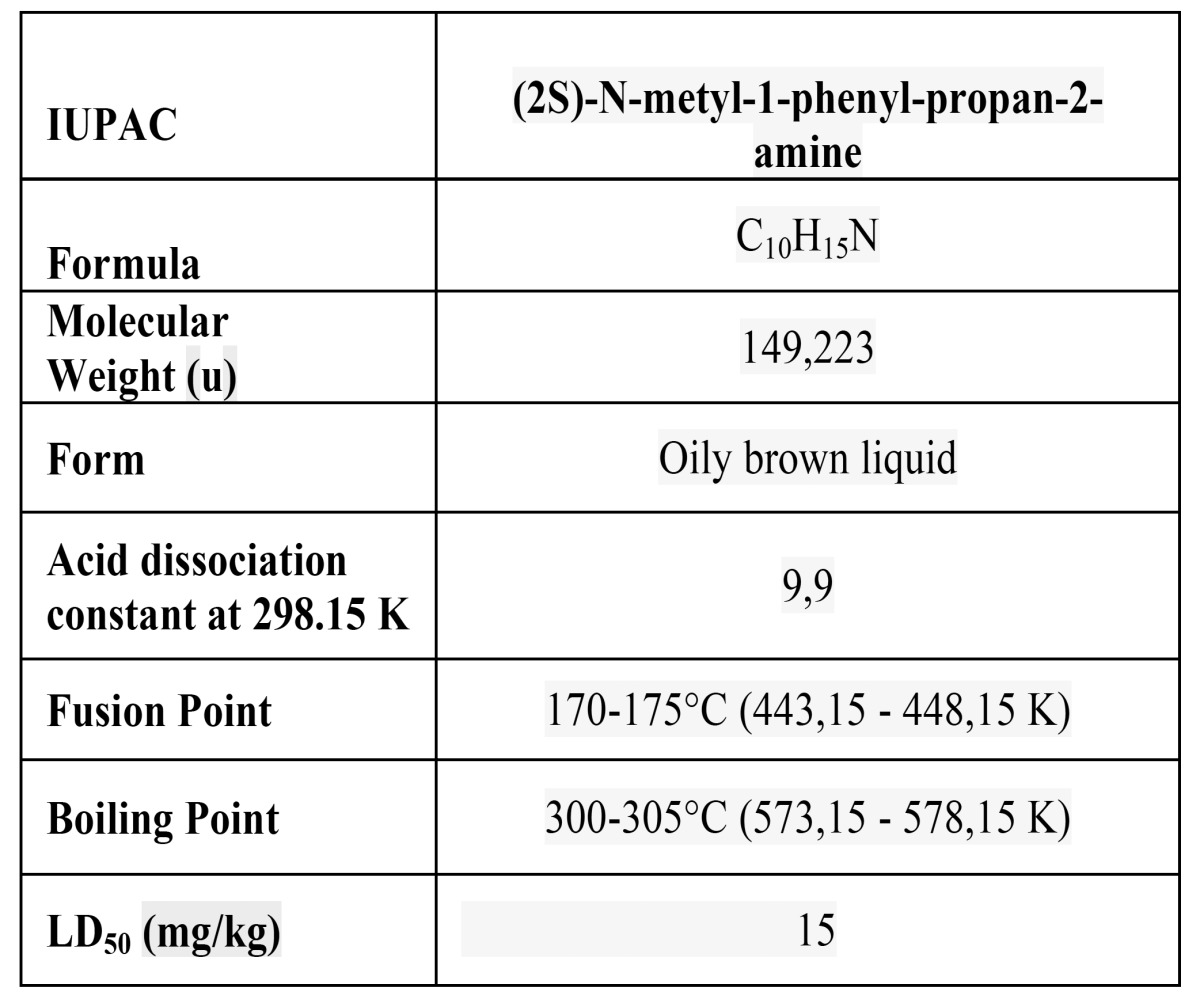
Chemical properties of methamphetamine (MA).

After oral administration maximum plasma concentrations are reached in 2 or 3 hours but the effects manifest after only 20 minutes. With intravenous injection maximum plasma concentrations occur within 2 to 4 minutes (7). 

MA is metabolized by microsomal enzymes in the liver, but chronic use does not increase their number. It is oxidized by part of the P-450 cytochrome 2D6 isoenzyme and glucuronidized into one active metabolite (amphetamine) and two inactive metabolites (nor-ephedrine and p-hydroxyl-nor-ephedrine) (8). Excreted through blood and kidneys, it has a half-life of 8 to 30 hours, according to the urinary pH. When it ranges from 6 to 8, MA is cleared in about 12 hours, independently of the administration route (7-9,10).

In habitual users MA markedly modifies the function of specific cerebral areas like the prefrontal cortex and the caudal nucleus, particularly the “nucleus accumbens”, that are linked to emotions and memory (5). You can develop a tolerance resulting from quick depletion of the availability of neurotransmitters which also contributes to the cause withdrawal crisis (10).

Short-term effects include euphoria, loquacity, over-excitement, insomnia, tremors, tachypnea and lack of appetite (10,11) while over the long-term the user manifests cognitive and emotional changes, violent behaviour, anxiety, paranoia, hallucinations, irritability and mood swings (5).

Overdose is associated with angina, dyspnoea, sweating, palpitations, nausea, vomiting, convulsions and hallucinations. More severe side effects are ventricular fibrillation, myocardial infarction, cardiovascular collapse and high temperature. If not treated promptly with urine acidification, you may have even death (2).

Typical withdrawal symptoms are depression, anxiety, fatigue and irrepressible desire for the drug. If MA and cocaine withdrawal are associated the patient may have suicidal and homicidal tendencies and a severe sleep disorder. MA use during pregnancy can cause miscarriage or premature birth and, in the newborn, hypotrophy, congenital abnormalities, delayed development and withdrawal symptoms (12).

Although amphetamine and its analogues were administered in the past to treat depression, attention deficit, obesity and narcolepsy, MA was always demonstrated to be too active and too dangerous. Even its analogues like for example methylphenidate causes side effects like hyperactivity, euphoria, loss of appetite, loquacity and increased libido (13) ( Table 3).

**Table 3:**
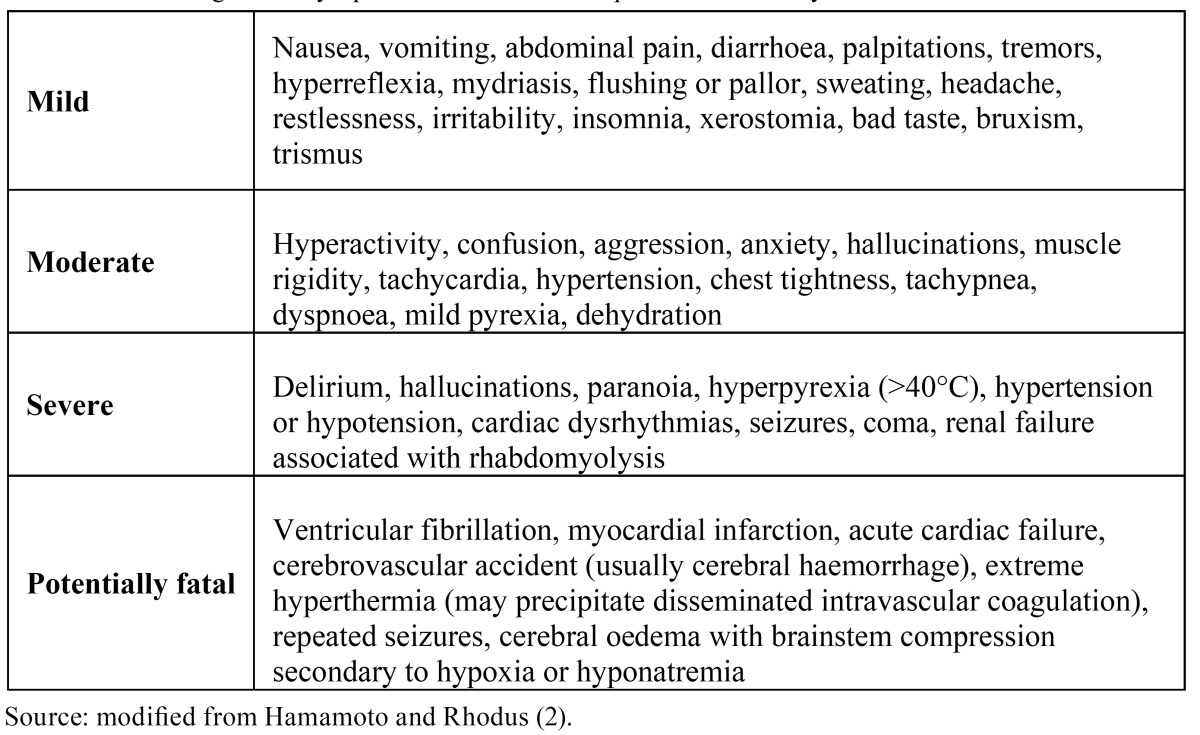
Clinical signs and symptoms of acute methamphetamine toxicity.

## Methamphetamine abuse and dental disease

Several reports have linked MA abuse to oral pathologies. “Meth mouth” is the general term for rampant caries like “early childhood caries” (14-17). Lesions are localized on all smooth buccal surfaces and on proximal interfaces of the front teeth (2,4,13,15,18) (see Fig.1). Teeth are described as “blackened, stained, rotting, crumbling or falling apart” (19) and “the typical pattern of decay involves the facial and cervical areas of both the maxillary and mandibular teeth with eventual progression to frank coronal involvement” (3).

Despite several clinical studies, the incidence of caries in MA abusers has only recently been reported as greater than in the general population. A pilot study comparing 18 MA users and 18 controls showed plaque and DMFT indices were much higher in users (20). In another report plaque, DMFS and dental calculus levels were significantly higher in 28 users than in 16 controls (21). In yet another investigation DMFS and S-OHI were significantly higher in a cohort of 58 young adult users than in the general population (22).One study observed that DMFT was significantly higher in 59 young adult prisoners who were MA users than in 40 control prisoners who were not and demonstrated that duration of use was the most significant predictor of “meth mouth” severity (23). In spite of these findings the high incidence of caries does not appear useful in distinguishing between users and nonusers (24).

“Meth mouth” is hypothesized to be caused by a combination of MA-related mental and physiological changes such as xerostomia (dry mouth), long periods of poor mouth hygiene, and frequent ingestion of sugary, fizzy drinks (2,3,14-16). Additional factors might be the acidic nature of MA (2-4,16) which, through contact with teeth after inhalation, might exert a caustic action (16).

Recent studies have provided evidence in support of some of these hypotheses.

1. Ravenel *et al*. observed no significant differences in saliva flow levels in 28 MA users and 16 controls but found saliva pH and buffer capacity tended to be lower in users, suggesting saliva quality played a key role in “meth mouth” (21). Some investigations confirmed the detrimental impact of long periods of poor oral hygiene and increased consumption of sugary drinks (20,22). In showing that “meth mouth” was similar in intravenous MA users Shetty *et al*. (19) refuted the hypothesis that MA exerted a corrosive or acidic effect on teeth. Rather surprisingly, Brown *et al*. (23) did not find concomitant alcohol abuse was a significant risk factor for “meth mouth” severity in 50 prisoners who were MA users, which seems to suggest the MA mechanism of action is different to other illegal substances.

2. The debate over whether xerostomia (dry mouth) is associated with MA abuse is still open, with some reports claiming dry mouth is present (2-4) and others denying it (22) on the basis of saliva flow analysis. Complaints of dry mouth might be subjective as 95% of 119 subjects in one study declared it was present (16) while 90% of 17 patients in another did not complain of it (22).

3. Excess dental wear is mainly due to teeth grinding (2,4,25) and possibly para-functional jaw activity, periodontal or temporomandibular disorders (2,25). In 43 MA users Richard and Brofeld (26) established an increased incidence in tooth wear, the severity of which varied with administration route as the most advanced tooth wear was detected in patients who sniffed MA. Shetty *et al*. (19) found tooth grinding (bruxism) or erosion in 22.3% of 301 patients (67 individuals).

The clinical signs of “meth mouth” do not differ from bad oral hygiene and dental destruction observed on other drugs abusers (e.g. heroin), but are faster to appear and more severe (22). One hypothesis is that the corrosive effect of Ma and its derivatives can occur locally because of their excretion through the crevicular fluid.

## Treating dental patients who are methamphetamine users

Most MA users who attend a dental clinic do not admit they are users when giving their case history for fear of being judged or reported to the police. Consequently dentists should be trained to recognize the main signs and symptoms of MA abuse. After obtaining as much information on the patient’s medical and dental history as s/he will provide, dental surgeons should observe whether the patient has skin lesions on the arm which indicate intravenous use of illegal drugs and ask about sudden fever of unknown origin which might suggest the same. Paranoid behaviour with mood swings, poor compliance and episodes of violence should be carefully assessed as they might also indicate addiction.

Dentists should bear in mind that risk of acquiring and transmitting blood-borne diseases like HIV and hepatitis B and C is increased in MA users because the MA-induced high may be associated with lack of inhibition of risky sexual (27).

General dental treatment for MA users should adhere to the following fundamental guidelines (2,3,11): if a patient shows signs of recent MA ingestion, only supportive treatment should be given; the team should be ready to implement safety measures for itself and other patients should the MA user exhibit paranoid or violent behaviour; MA users who are “high” should not receive any dental treatment whatsoever until at least 6 hours have elapsed since the drug was taken, because sympathomimetic effects at this stage are associated with a very high risk of myocardial ischemia and arrhythmia; should dental treatment be needed, local anaesthetics should never contain adrenalin or noradrenalin because they can potentiate the sympathomimetic response to MA, which could raise blood pressure excessively and cause a cerebral vascular accident or myocardial infarction; a general anesthesia or sedation can cause sudden death in an MA user; since an increased respiratory rate (tachypnea) can lead to depressed respiration, the patient will require 100% oxygen and frequent heart rate and blood pressure checkups; heart and lung resuscitation apparatus should be readily available in case the patient suffers a cardiovascular collapse.

## Short notes of epidemiology

MA production is greatest in the Far East and Southeast Asia (China, the Philippines, Myanmar and Thailand), followed by North and Central America (the USA, Canada and Mexico) (17). MA appears to be mainly produced in Europe in the Czech Republic but most is destined for the domestic market although some reaches Germany, Austria and Slovakia (28).

**Figure 2:**
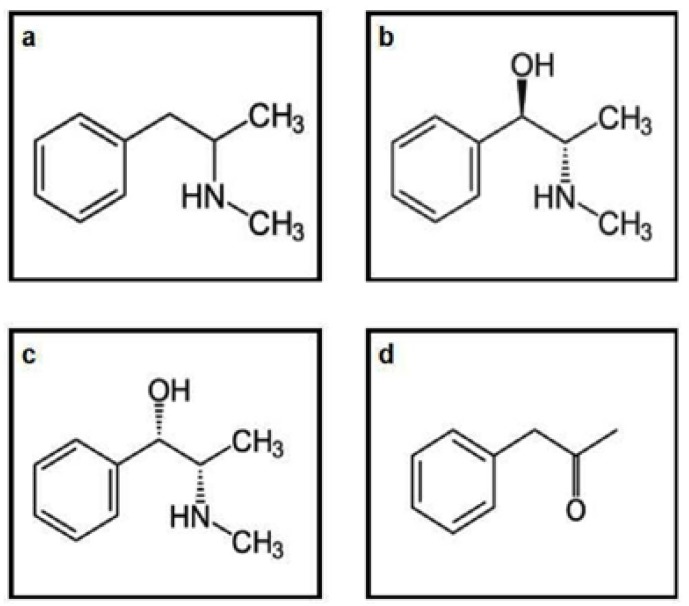
Formula and molecular structures of methamphetamine (panel a) and its precursors ephedrine (panel b), pseudoephedrine (panel c) and 1-phenyl-2-propanone (panel d).

Ephedrine, pseudoephedrine and 1-phenyl-2-propanone (P-2-P o BMK) (Fig. 2) are the main MA precursors. MA is produced illegally by reducing ephedrine or pseudo ephedrine with iodine, hypophosphite or ammoniac ions while legal pharmaceutics are produced by reductive amination of P-2-P. Since ephedrine or pseudo ephedrine are contained in drugs for human use they are excluded from the 111/2005 CE regulation which legislates for trade in drug precursors between the EU and non-EU countries.

Major problems with MA use have been referred by many countries including the USA, Southeast Asia, the Pacific and Africa (28). The American Dental Association (17) confirmed findings in the 2010 National Survey on Drug Use and Health, by stating that although MA use appears to be falling it is still a matter of concern.

MA appears to be less extensively used in Europe than in the USA. Abuse show to peak in 2005-2007 and then decline in central (Czech Republic, Slovakia, Hungary and Slovenia) and northern (UK, Denmark, Norway, Finland and Sweden) Europe. MA abuse is negligible in southern European countries and predominates among young adults (28).

## References

[1] Mariotti KC, Rossato LG, Fröehlich PE, Limberger RP (2013). Amphetamine-type medicines: a review of pharmacokinetics, pharmacodynamics and toxicological aspects. Curr Clin Pharmacol.

[2] Hamamoto DT, Rhodus NL (2009). Methamphetamine abuse and dentistry. Oral Dis.

[3] Goodchild JH, Donaldson M (2007). Methamphetamine abuse and dentistry: a review of the literature and presentation of a clinical case. Quintessence Int.

[4] Klasser GD, Epstein J (2005). Methamphetamine and its impact on dental care. J Can Dent Assoc.

[5] Nordahl TE, Salo R, Leamon M (2003). Neuropsychological effects of chronic methamphetamine use on neurotransmitters and cognition:a review. J Neuropsychiatry Clin Neurosci.

[6] Sulzer D, Sonders MS, Poulsen NW, Galli A (2005). Mechanisms of neurotransmitter release by amphetamines :a review. Prog Neurobiol.

[7] Schepers RJ, Oyler JM, Joseph RE, Cone EJ, Moolchan ET, Huestis MA (2003). Methamphetamine and amphetamine pharmacokinetics in oral fluid and plasma after controlled oral methamphetamine administration to human volunteers. Clin Chem.

[8] Olkkola KT, Ahonen J (2001). Drug interactions. Curr Opin Anaesthesiol.

[9] Shimosato K, Tomita M, Ijiri I (1986). Urinary excretion of p-hydroxylated methamphetamine metabolites in man. I. A method for determination by high-performance liquid chromatography-electrochemistry. Arch Toxicol.

[10] Cook CE, Jeffcoat AR, Sadler BM, Hill JM, Voyksner RD, Pugh DE (1992). Pharmacokinetics of oral methamphetamine and effects of repeated daily dosing in humans. Drug Metab Dispos.

[11] Lee CY, Heffez LB, Mohammadi H (1992). Crystal methamphetamine abuse: a concern to oral and maxillofacial surgeons. J Oral Maxillofac Surg.

[12] Dixon SD, Bejar R (1989). Echoencephalographic findings in neonates associated with maternal cocaine and methamphetamine use: incidence and clinical correlates. J Pediatr.

[13] Rhodus NL, Little JW (2005). Methamphetamine abuse and ''meth mouth''. Northwest Dent.

[14] Howe AM (1995). Methamphetamine and childhood and adolescent caries. Aust Dent J.

[15] Shaner JW (2002). Caries associated with methamphetamine abuse. J Mich Dent Assoc.

[16] McGrath C, Chan B (2005). Oral health sensations associated with illicit drug abuse. Br Dent J.

[17] American Dental Association (2005). For the dental patient. Methamphetamine use and oral health. J Am Dent Assoc.

[18] Eramo S, Baldi M, Marci MC, Monaco A (2003). Histopathological and therapeutical aspects of cervical lesions. Minerva Stomatologica.

[19] Shetty V, Mooney LJ, Zigler CM, Belin TR, Murphy D, Rawson R (2010). The relationship between methamphetamine use and increased dental disease. J Am Dent Assoc.

[20] Morio KA, Marshall TA, Qian F, Morgan TA (2008). Comparing diet, oral hygiene and caries status of adult methamphetamine users and nonusers: a pilot study. J Am Dent Assoc.

[21] Ravenel MC, Salinas CF, Marlow NM, Slate EH, Evans ZP, Miller PM (2012). Methamphetamine abuse and oral health: a pilot study of "meth mouth". Quintessence Int.

[22] Brown C, Krishnan S, Hursh K, Yu M, Johnson P, Page K (2012). Dental disease prevalence among methamphetamine and heroin users in an urban setting: a pilot study. J Am Dent Assoc.

[23] Brown RE, Morisky DE, Silverstein SJ (2013). Meth mouth severity in response to drug-use patterns and dental access in methamphetamine users. J Calif Dent Assoc.

[24] Cretzmeyer M, Walker J, Hall JA, Arndt S (2007). Methamphetamine use and dental disease: results of a pilot study. J Dent Child (Chic).

[25] Curtis EK (2006). Meth mouth: a review of methamphetamine abuse and its oral manifestations. Gen Dent.

[26] Richards JR, Brofeldt BT (2000). Patterns of tooth wear associated with methamphetamine use. J Periodontol.

[27] Shoptaw S, Reback CJ (2006). Associations between methamphetamine use and HIV among men who have sex with men: a model for guiding public policy. J Urban Health.

[28] Griffiths P, Mravcik V, Lopez D, Klempova D (2008). Quite a lot of smoke but very limited fire: the use of methamphetamine in Europe. Drug and Alcohol Review.

